# Endovis Nail versus Dynamic Hip Screw for Unstable Pertrochanteric Fractures: A Feasibility Randomised Control Trial including Patients with Cognitive Impairment

**DOI:** 10.3390/jcm12134237

**Published:** 2023-06-23

**Authors:** George Kleftouris, Theodoros H. Tosounidis, Michalis Panteli, Martin Gathen, Peter V. Giannoudis

**Affiliations:** 1 Academic Department of Trauma and Orthopaedics, School of Medicine, University of Leeds, Leeds LS2 9LU, UKpgiannoudi@aol.com (P.V.G.); 2 Department of Orthopaedic Surgery, University Hospital of Heraklion, 71500 Heraklion, Greece; 3 Department of Orthopaedics and Trauma Surgery, University Hospital of Bonn, 53127 Bonn, Germany; 4 NIHR Leeds Biomedical Research Center, Chapel Allerton Hospital, Leeds LS7 4SA, UK

**Keywords:** hip fractures, unstable, OTA/AO 31-A2 fractures, cognitive impairment, feasibility study

## Abstract

A prospective, feasibility, randomised study was performed to compare intramedullary versus extramedullary fixation of unstable pertrochanteric fractures and to assess the feasibility of including patients with dementia. From July 2016 to November 2017, 60 consecutive patients with an unstable pertrochanteric (OTA/AO 31-A2) fracture were randomized to either receive a short cephalomedullary nail (Endovis EBA^2^, Citieffe) or a dynamic hip screw (DHS, Zimmer Biomet). Primary feasibility measures included randomisation, recruitment, and retention rates. Secondary outcomes included peri-operative parameters, patient-reported outcomes and radiographic outcomes. Patients were followed-up at two, four, and twelve weeks. There was no difference in the randomisation rate between patients with and without cognitive impairment. Significantly more patients without cognitive impairment attended the 12-week follow-up. The overall recruitment rate was 0.9 patients per week. Patients treated with the nail had less pain at 2 weeks and less neck collapse, medialisation, and leg shortening at all time points. The rest of secondary outcomes were similar. Patients with dementia can successfully be enrolled in a randomised trial on hip fractures. Patients treated with the Endovis nail had lower levels of pain at two weeks and better radiographic outcomes.

## 1. Introduction

Because of the growing ageing population, the incidence of hip fractures continues to increase [1]; in the UK, the number of hip fractures increased from 36,500 in 2009 to 65,900 in 2018 [2]. These injuries have a great impact, not only on individuals, but also on the healthcare system [2].

Dementia affects 7.1% of people over 65 in the UK and the number of people with dementia is estimated to exceed two million in 2050 [3]. More importantly, people with dementia have at least a twice higher risk of sustaining a hip fracture than people without dementia [4].

Almost half of all hip fractures are extracapsular and can be stabilised with various fixation devices [5]. Currently, the dynamic hip screw (DHS) is the preferred implant for stable (OTA/AO 31-A1) intertrochanteric fractures [6]. In contrast, intramedullary nailing is the recommended implant for subtrochanteric or reverse oblique (OTA/AO 31-A3) fractures [7]. Controversy still remains on optimal fixation devices for unstable pertrochanteric fractures (OTA/AO 31-A2) [8].

Although a third of patients with hip fractures have a degree of cognitive impairment, eight out of ten randomised control trials (RCTs) on hip fractures exclude patients with dementia [9]. At the same time, there is a paucity of feasibility studies in the literature that assess different fracture fixation devices and include patients with cognitive impairment. Consequently, the purpose of this study was as follows: (1) To assess the feasibility of performing an RCT including patients with cognitive impairment and (2) to compare intramedullary versus extramedullary fixation for unstable pertrochanteric fractures (OTA/AO 31-A2).

## 2. Materials and Methods

### 2.1. Ethics Approval

The study was performed in line with the guidelines of the Good Medical Practice and the Declaration of Helsinki. Approval was granted by the local Research Ethics Committee (REC: 15/YH/0440) and the trial was registered at ClinicalTrials.gov (NCT02788994). The results of this study were reported according to the CONSORT 2010 statement for feasibility studies (CONSORT checklist available upon request).

### 2.2. Study Population

Between July 2016 and November 2017, all patients over 55 years who presented to our institution with a proximal femur fracture (OTA/AO 31-A2) were screened for eligibility [10]. Exclusion criteria included pathological fractures; patients unable to mobilize at least six meters prior to injury; and patients with a new diagnosis of acute myocardial infarction, stroke, or multi-organ failure.

Cognitive status was assessed using the Abbreviated Mental Test Score (AMTS). Patients with AMTS < 8 were considered to be cognitively impaired [11]. For these patients, consent was obtained by personal or nominated consultee in accordance with the Mental Capacity Act 2005 [12]. If patients had an AMTS < 8 upon admission and regained capacity during the hospital admission, they were re-consented. Patients were randomised with a 1:1 ratio to either the nail (EBA^2^, Citieffe) or the DHS (Zimmer Biomet) group. Randomisation was stratified by cognitive status (AMTS < 8 or AMTS ≥ 8) in permuted block sizes of six, using an online randomisation system provided by King’s Clinical Trial Unit, London, UK. The operating surgeon and research team were not blinded to the treatment group for obvious reasons. The patients were also considered unblinded as they could guess the type of operation they had due to the number of incisions.

All of the procedures were performed by senior trauma surgeons according to the manufacturer’s operative technique. Patients were managed by a multi-disciplinary team, according to our hospital standardized peri-operative protocol. Patients were followed-up at two, four, and twelve weeks. A telephone call was made at twelve months to assess for any late adverse events.

### 2.3. Feasibility Assessment

To assess the feasibility of an RCT including patients with dementia, we assessed the randomisation rate, recruitment rate, and retention rates. The randomisation rate was calculated as the proportion of eligible patients randomised to the study. The recruitment rate was calculated as the average number of patients recruited per week. The retention rate was calculated as the proportion of patients who attended week four and week twelve follow-up visits.

### 2.4. Secondary Outcomes

Baseline characteristics included patient demographics, comorbidities, level of mobility, place of residence, pre-operative pain Numeric Rating Scale (NRS) [13], and functional and quality of life questionnaires. Cognitively intact patients completed the Lower Extremity Measure (LEM) [14] and the London Handicap Scale (LHS) [15]; cognitively impaired patients completed the Dementia Quality of Life measure (DEMQOL) [16]. Peri-operative outcomes included duration of surgery, blood loss (ml of blood in suction tube and weight (in gr) of surgical gauzes used), post-operative haemoglobin drop, blood transfusion requirements within the first two weeks, time until ready for discharge (defined as the number of days until patient deemed fit for discharge by the medical team and the physiotherapists), overall length of stay, analgesia requirements within the first two weeks (morphine equivalent dose), and mortality rates at three and twelve months.

At each follow-up visit, mobility was assessed using the Timed Up and Go (TUG) test [17]; patients were asked to rise from a chair, walk three meters using a frame with wheels, turn around, walk back, and sit down. The test was performed three times where possible and the best time was recorded. The assessor was blinded to the randomisation group.

Anteroposterior pelvis and lateral hip radiographs were taken at each visit. The tip–apex distance (TAD) was measured as described by Baumgartner (for patients treated with a nail, the inferior screw was used to calculate the TAD) [18]. Neck collapse was measured as the distance from the medial edge of the femoral head to the lateral cortex of the femur [19]. Shortening was measured as the distance of the lesser trochanter from the trans-ischial line [20]. Medialisation was measured as the medial displacement of the distal fracture in relation to the proximal fragment [21]. Fracture union was assessed using the Radiographic Union Score for Hip (RUSH) at 12 weeks [22]. All radiographs were calibrated prior to measurements.

### 2.5. Sample Size

A sample size of 60 patients (*n* = 30 per group) was deemed sufficient for this feasibility study. This is consistent with a recent simulation study on sample size requirements; according to the simulation analysis, even with as little as 10 patients per group, estimates of standard deviation are sufficiently accurate to yield sample sizes for planned full trials at least 75% of the time [23]. The TUG test was used as a guide to power a full-scale trial.

### 2.6. Statistical Analysis

Statistical analysis was performed using the SPSS software (Version 23; SPSS Inc., Chicago, IL, USA). Feasibility outcomes were reported as percentages. Descriptive statistics were presented as mean ± SD when data were normally distributed or as median (range) when not normally distributed. An unpaired, two-sided *t*-test was used to compare normally distributed data and Mann–Whitney U test was used for non-parametric data. Number of patients and percentages were compared using Chi-square test and Fisher exact test. Level of significance was at <0.05. An intention to treat the approach was used. Patients withdrawn were not replaced and no imputation of missing data was attempted.

## 3. Results

### 3.1. Feasibility Assessment

Out of the 86 eligible patients, 60 were randomised in the trial with a randomisation rate of 69.8%. There was no difference in the randomisation rate between patients with and without dementia (randomisation rate 68.8% and 70.4%, respectively, *p* = 0.874). The recruitment rate was 0.9 patients per week.

**Figure 1:**
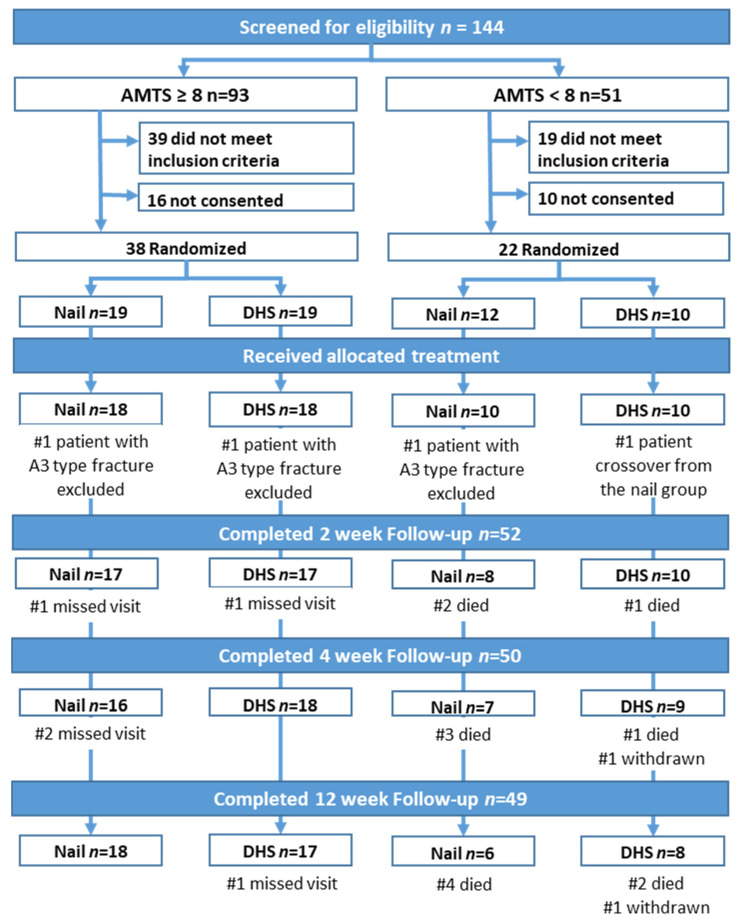
Consort flow diagram of patients as per the randomised treatment.

Three patients (two from the nail group and one from the DHS group) were found to have an AO/OTA 31A3 fracture when positioned on the traction table and were excluded from further analysis. One patient from the nail group received a DHS. Patient flow at each stage of the trial is shown in Figure 1.

The overall retention rate was 87.7% (50/57) at 4 weeks and 86% (49/57) at 12 weeks. Although there was no significant difference in retention rate at 4 weeks between patients without and with dementia (94.4% vs. 76.2%, *p* = 0.088), more patients without dementia completed 12 weeks of follow-up (97.2% vs. 66.7%, *p* = 0.003).

The main barriers to recruitment included patients declining participation, patients not speaking English, patients too anxious to decide, lack of time to consent, and presentations during weekends or bank holidays. The main barriers to follow-up included deaths and hospital transportation issues.

### 3.2. Secondary Outcomes

Baseline demographics and patient characteristics were comparable in the two treatment groups (Table S1 in Supplementary Materials).

#### 3.2.1. Peri-Operative Outcomes

There were no differences between the nail and the DHS group in the duration of surgery, blood loss, length of stay, and analgesia requirements. Readiness for discharge was significantly shorter (11 vs. 20 days, *n* = 18, *p* = 0.029) in patients with dementia treated with a nail. Although the haemoglobin drop (27.0 vs. 29.8 g/L) and transfusion requirements (44.8% vs. 53.6%) were higher in the DHS group, these differences did not reach statistical significance (*p* > 0.4) (Table 1).

#### 3.2.2. TUG Test 

The overall completion rates of the TUG test at two, four, and twelve weeks were 63.5%, 84%, and 88.6%, respectively. Although there was no significant difference in the patients’ ability to perform the test when patients were split by treatment received, significantly fewer patients with dementia were able to perform the TUG test at 2 weeks (38.9% vs. 76.5%, *p* = 0.014); this difference was not observed at 4 and 12 weeks. There was no difference in TUG times between the nail and DHS group at all time points (Table 2).

#### 3.2.3. Radiographic Results 

The average tip−apex distance was higher in the nail group (18.2 mm vs. 14.9 mm, *p* = 0.044). Fractures treated with DHS had significantly higher neck collapse (*p* ≤ 0.001), shortening (*p* ≤ 0.001), and medial displacement (*p* ≤ 0.03) at all time points. Both groups had a similar RUSH score at 12 weeks (22.9 vs. 24.5, *p* = 0.277) (Table 3).

#### 3.2.4. Patient Reported Outcome Scores 

There was no difference in LEM, LHS, and DEMQOL between the nail and DHS group at 2, 4, and 12 weeks. Patients treated with a nail had significantly lower levels of pain than patients treated with DHS at 2 weeks (pain NRS, 5 versus 7.5, *p* = 0.003); this difference did not reach statistical significance at 4 and 12 weeks, respectively (Table 4).

#### 3.2.5. Complications and Mortality (Table 5)

The overall incidence of complications was similar in the two groups (nail: 31% vs. DHS: 28.6%). There was only one orthopaedic-related complication that occurred in the nail group. One patient sustained an undisplaced fracture at the level of the distal cephalic screw at week 5. He was treated non-operatively with assisted mobilization. The patient fully recovered by the final review at week 12.

There was no difference in mortality between the treatment groups at 2, 4, and 12 weeks and at 12 months (Table 5). When compared as per the AMTS group, patients with dementia had statistically significant higher mortality than patients without dementia at 2 (14.3% vs. 0%, *p* = 0.046), 4 (19% vs. 0%, *p* = 0.015), 12 weeks (28.6% vs. 0%, *p* = 0.002) and at 1 year (50% vs. 11.1%, *p* = 0.003) (Table S2 in Supplementary Materials).

**Table 5:** Complications and mortality rates.

	**Nail *n* = 29**	**DHS *n* = 28**	***p*** **-Value**
**Total complications, *n***	11	11	
Patients affected, *n* (%)	9 (31.0%)	8 (28.6%)	1.000
**Medical complications, *n***	10	11	
Patients affected, *n* (%)	8 (27.6%)	8 (28.6%)	1.000
**Orthopaedic complications, *n***	1	-	
Patients affected, *n* (%)	1 (3.4%)	-	1.000
Periprosthetic fracture	1	-	
**Mortality rates, *n* (%)**			
2 weeks	2 (7.9%)	1 (3.6%)	1.000
4 weeks	3 (10.3%)	1 (3.6%)	0.612
12 weeks	4 (13.8%)	2 (7.1%)	0.670
1 year	8 (28.6%)	6 (21.4%)	0.758

## 4. Discussion

This study shows that although it is possible to recruit and to enrol patients with cognitive impairment, it was more likely for these patients not to complete the study, mainly due to mortality. It was also noted that not all of the patients were able to complete the TUG test even at 12 weeks and, moreover, TUG times varied widely and as a result, comparisons between the groups were difficult. With regard to the type of fixation, patients treated with the Endovis nail had lower levels of pain at two weeks and overall better radiographic outcomes.

Although recruiting and enrolling patients with dementia in this RCT was not an issue, a significantly lower proportion of patients with dementia attended the 12-week follow-up (97.2% vs. 66.7%). This means that outcome measures will need to be assessed early in the follow-up if patients with dementia are to be included. Outcome measures assessed after 12 weeks may be more appropriate only for patients without dementia due to the high attrition rates in patients with dementia.

Other studies have also reported low percentages of patients able to perform the TUG test [24,25]. In this study, only 63.5% of all patients were able to perform the test at 2-weeks and this percentage increased to 84% and 88.6% at four and twelve weeks, respectively. The big variability in TUG times recorded in this study reflects the high heterogeneity in walking ability in this patient group; however, this could also be due to the lack of a pre-specified endpoint. After a pre-specified endpoint, the test should be terminated and the patient deemed unable to perform the test. High variability of an outcome measure makes comparisons between two groups difficult. For all these reasons, the TUG test was not a good physical outcome measure to assess functional mobility in this patient group and thus we would not recommend it for similar future trials. An alternative physical outcome measure would ideally be easier for patients to perform, have a clear endpoint, and ideally a value (e.g., zero) for patients who are unable to perform it. Such tests could be the 2-min walk test and the 30 seconds sit to stand test. However, neither of these tests have been validated in patients with hip fractures and further work is required before they can be used in a future trial. Similar to this study, other trials have reported a low percentage of patients able to complete the TUG test and also comparable times between the nail and the DHS groups [24,25,26].

In this study, it was found that patients treated with a nail had lower levels of pain at 2 weeks. However, this difference was not apparent beyond 4 weeks. These results are consistent with results from other studies [27,28,29]. This finding can be explained by the fact that intramedullary nailing is a percutaneous procedure sparing extensive soft tissue dissection associated with muscle damage, bleeding, and subsequent scarring. We recognize, however, that pain is a difficult variable to assess, not only because it is a subjective feeling, but also because patients with cognitive impairment may not be able to express it accurately. For this reason, we also recorded analgesia requirements. Although patients treated with DHS had higher analgesia requirements, this difference did not reach statistical significance. This may be due to the small size of the trial and a type II statistical error.

In terms of intra-operative blood loss and blood transfusion requirements, one would expect the nailing group to have less blood loss and transfusion requirements compared with the DHS group. A recent meta-analysis on A2 type fractures confirmed this [30]. We found that there was a trend for higher intraoperative blood loss, higher blood transfusion requirements, and higher haemoglobin drop in the DHS group, but these differences were not statistically significant. As above, this could also be due to a type II statistical error.

In terms of length of hospital stay, similar to other studies, there was no significant difference between the nail and the DHS group [8,28,31,32]. However, patients with dementia in the nail group were deemed ready for discharge significantly earlier than patients with dementia in the DHS group. We included readiness for discharge because apart from medical factors, social factors may prolong the hospital stay and thus this can mask a potential difference between the groups. Nevertheless, interpretation of any results on readiness of discharge has to be very careful at this stage because these findings should ideally be confirmed by a full-scale trial.

**Figure 2:**
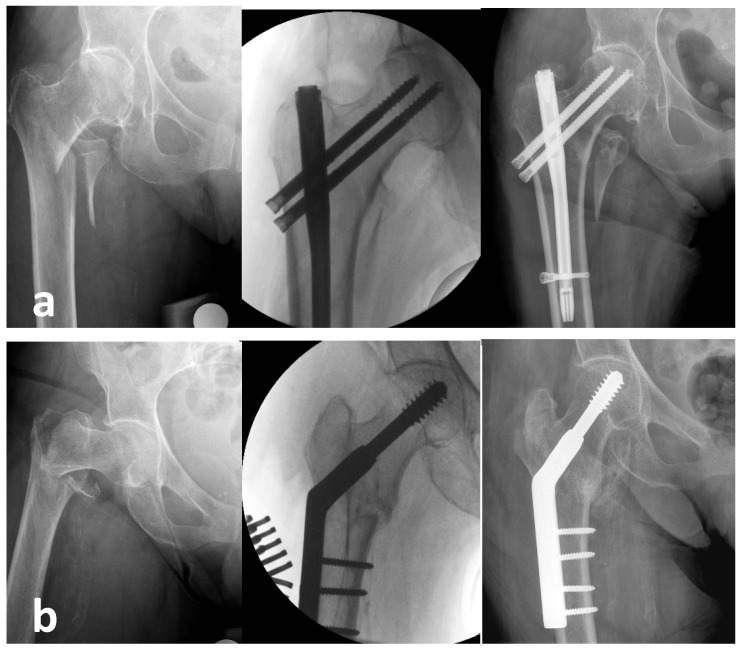
Assessment of fracture healing. (**a**) Pre-operatively, intraoperatively, and at 12 weeks for a patient treated with a nail. (**b**) Pre-operatively, intraoperatively, and at 12 weeks for a patient treated with DHS.

Similar to other studies, patients treated with a nail had significantly better radiographic outcomes than patients treated with DHS [26,32,33]. However, better radiographic outcomes have not translated into better functional outcomes, which likely reflects poor physical function in people with hip fractures [25]. Another reason for the lack of correlation between radiographic and functional results could be due to the fact that the difference of the radiographic parameters between the nail and the DHS group is not clinically significant. For valuable conclusions in a clinical study, the results need to be statistically but also clinically significant. With regard to fracture healing, there was no difference in the RUSH score between the two groups (Figure 2).

Similar to other RCTs and meta-analyses, the incidences of medical complications were high and comparable in both groups [25,29,30,33]. There was only one local orthopaedic related complication that was treated non-operatively and did not affect the overall recovery of the patient. Although there was no difference in mortality rates between the nail and the DHS group, there was a statistically significant difference in mortality between patients with and without dementia. Several other studies have shown similar results with no difference in mortality between intramedullary and extramedullary implants [34]. Dementia is a well-established risk factor for a poor prognosis in patients with hip fractures and increased rates of mortality [35].

The main strength of this study is the inclusion of patients with dementia in an RCT and reporting outcome measures specifically for this group. Secondly, this study included only OTA/AO 31-A2 fractures; inclusion of both stable (type A1) and unstable (type A2) fractures can potentially mask any difference between intramedullary and extramedullary fixation. Thirdly, this study used different patient-reported outcomes, depending on patient’s cognitive status; patients with dementia are frequently unable to complete the same questionnaires as patients who are cognitively intact.

The main limitation of this study was the small number of participants and high level of attrition among patients with cognitive impairment. The small number of participants was due to the nature of this trial, which was a feasibility study; therefore it aimed to recruit a small number of patients (in this case 30 participants per group). The high level of attrition among patients with dementia made a comparison of variables within this group difficult; however, this was an important feasibility finding for future trials (i.e., patients with cognitive impairment are less likely to complete the study than patients without cognitive impairment). The follow-up was determined to three months for two reasons: firstly, it has been reported previously that improvement in mobility in patients with hip fractures occurs by three months, and secondly, as this was a feasibility study, it would be quicker to complete it with a three-month follow-up than a longer one [36]. The TUG test was not a suitable physical assessment measure for this group of patients and this can be considered as another limitation of this study; no sample size calculation was possible.

In conclusion, it is feasible to conduct an RCT on hip fractures including patients with cognitive impairment. Patients with dementia should not be excluded from randomised trials on hip fractures in the future. Fixation with the EBA^2^ nail resulted in better radiographic outcomes and lower levels of pain in the early post-operative period. Further work is required to identify alternatives to TUG test physical assessment measures.

## Figures and Tables

**Table 1:** Peri-operative results.

Peri-Operative Results	Nail (*n* = 29)	DHS (*n* = 28)	Mean Difference (95% Confidence Interval)	*p*-Value
Duration of surgery (min), mean ± SD	46.2 ± 12.5	48.9 ± 13.5	−2.7 (−9.6, 4.2)	0.440
Blood loss (mls), mean ± SD	83.2 ± 66.5	126.7 ± 102.6	−43.5 (−90.4, 3.4)	0.068
Blood loss (grams), mean ± SD	76.6 ± 65.8	79.1 ± 33.6	−2.5 (−47.6, 42.7)	0.911
Hb drop (g/L), mean ± SD	27 ± 16.3	29.8 ± 13.4	−2.8 (−10.8, 5.1)	0.480
Blood transfusion requirements, *n* (%)	13 (44.8%)	15 (53.6%)	-	0.600
Blood transfusion units, mean ± SD	1.9 ± 0.3	2.0 ± 0.7	−0. 1 (−0.5, 0.2)	0.697
Days till ready for discharge (days), median (range)				
All patients	9 (2–27)	12 (1–47)		0.148
AMTS < 8	11 (2–15)	20 (10–47)		0.029
AMTS ≥ 8	7 (4–27)	9 (1–25)		0.656
Length of stay (days), median(range)	21 (9–72)	22 (10–55)		0.562
Morphine equivalent dose (mg), median(range)	72.25 (0–564)	91 (3.75–1400)		0.203

**Table 2:** Timed UP and Go test results. Proportion of patients able to perform the TUG test according to treatment received and according to cognitive status. TUG: Timed Up and Go; AMTS: Abbreviated Mental Test Score.

Able to Perform the TUG Test, *n* (%)	Nail	DHS	*p*-Value
2 weeks	14/26 (53.8%)	19/26 (73.1%)	0.150
24 weeks	21/23 (91.3%)	21/27 (77.8%)	0.193
12 weeks	18/20 (90%)	21/24 (87.5%)	0.795
**Able to Perform the TUG Test; *n*/N (%)**	**AMTS ≥ 8**	**AMTS < 8**	
2 weeks	26/34 (76.5%)	7/18 (38.9%)	0.014
4 weeks	29/34 (85.3%)	13/16 (81.3%)	0.699
12 weeks	30/32 (93.8%)	9/12 (75%)	0.081
**TUG Times (s), Median (Range)**	**Nail**	**DHS**	
2 weeks	75 (20–177)	120 (20–295)	0.585
4 weeks	59 (16–381)	51 (13–329)	0.669
12 weeks	37.5 (16–229)	31 (14–119)	0.317

**Table 3:** Radiographic results. TAD: tip−apex distance.

Radiographic Results	Nail (*n* = 29)	DHS (*n* = 28)	Mean Difference (95% Confidence Interval)	*p*-Value
TAD (mm), mean ± SD	18.2 ± 6.8	14.9 ± 4.3	3.2 (0.1, 6.4)	0.044
Neck collapse (mm), mean ± SD				
Week 2	1.19 ± 2.2	9.5 ± 8.6	−8.3 (−12.2, −4.4)	<0.001
Week 4	3.4 ± 4.0	10.6 ± 8.1	−7.2 (−11.1, −3.3)	0.001
Week 12	2.9 ± 3.5	12.2 ± 9.4	−9.4 (−14.0, −4.8)	0.001
Medial displacement >5 mm, *n* (%)				
2 weeks	4 (16%)	15 (53.6%)		0.009
4 weeks	3 (13%)	16 (61.5%)		0.001
12 weeks	2 (9.5%)	15 (62.5%)		<0.001
Shortening (mm), mean ± SD				
Week 2	5.1 ± 5.7	9.8 ± 9	−4.7 (−8.9, −0.4)	0.032
Week 4	5.2 ± 6	12.1 ± 9.5	−7 (−11.5, −2.4)	0.004
Week 12	6.8 ± 7	12.2 ± 8.6	−5.4 (−10.2, −0.6)	0.029
RUSH 12 weeks, mean ± SD	22.9 (5.7)	24.5 (4.4)	−1.7 (−4.8, 1.4), *n* = 45	0.277

**Table 4:** Patient-reported outcome scores. LEM: Lower Extremity Measure; LHS: London Handicap Scale; DEMQOL: Dementia Quality of Life.

Patient-Reported Outcome Scores	Nail (*n* = 29)	DHS (*n* = 28)	*p*-Value
LEM, median (range)			
Pre-injury	59.5 (31.3–100)	69.2 (48.3–100)	0.208
2 weeks	41.7 (9.8–73.3	31.3 (11.6–87.5)	0.403
4 weeks	48.2 (8.62–77.4)	43.1 (14.3–78.6)	0.595
12 weeks	61.3 (11.2–84.8)	54.2 (5–89.8)	0.903
LHS, median (range)			
Pre-injury	0.66 (0.53–1.00)	0.74 (0.54–1.00)	0.402
2 weeks	0.58 (0.42–0.79)	0.56 (0.36–0.83)	0.403
4 weeks	0.62 (0.42–0.83)	0.65 (0.47–0.83)	0.761
12 weeks	0.69 (0.28–0.93)	0.67 (0.45–1.0)	0.804
DEMQOL, median (range)			
Pre-injury	87.0 (69.0–95.0)	73.6 (40.0–108.0)	0.350
2 weeks	89.0 (66.0–106.0)	75.5 (48.0–112.0)	0.714
4 weeks	84.7 (61.0–103.0)	88.5 (66.0–112.0)	0.671
12 weeks	95.0 (84.0–111.0)	85 (60–107)	0.305
DEMQOL-carer, median (range)			
Pre-injury	99.2 (84.0–117.0)	97.6 (64.0–115.0)	0.642
2 weeks	98.0 (90.0–106.0)	92.0 (77.0–115.0)	0.599
4 weeks	103.0 (93.0–111.0)	92.9 (79.0–113.0)	0.464
12 weeks	98.5 (65.0–114.0)	86.0 (73.0–110.0)	0.507
Pain NRS, median (range)			
Pre-op	8.0 (0.0–10.0)	8.0 (0.0–10.0)	0.967
2 weeks	5.0 (0.0–9.0)	7.5 (2.0–10.0)	0.003
4 weeks	5.0 (0.0–8.0)	7.0 (0.0–10.0)	0.074
12 weeks	3.5 (0.0–8.0)	2.0 (0.0–9.0)	0.795

## Data Availability

The data presented in this study are available upon request from the corresponding author.

## Supplementary Materials

The following supporting information can be downloaded at: https://www.mdpi.com/article/10.3390/jcm12134237/s1. Table S1: Patients baseline demographics. Table S2: Mortality rates as per AMTS group at 2, 4, 12, weeks and at 1 year.
