# Supplementary material for: Endovis Nail versus Dynamic Hip Screw for Unstable Pertrochanteric Fractures: A Feasibility Randomised Control Trial including Patients with Cognitive Impairment
Source: J Clin Med. 2023 Jun 23;12(13):4237. doi: 10.3390/jcm12134237 (PMC10342809; doi:10.3390/jcm12134237)
Supplement: Supplementary file 1 [file jcm-12-04237-s001.zip › jcm-2428540-supplementary.pdf]

Supplementary Materials:

Table S1: Patients baseline demographics; ASA: American Society of Anesthesiologist score, AMTS: Abbreviated Mental Test Score, LEM: Lower Extremity Measure, LHS: London Handicap Scale, DEMQOL: Dementia Quality of Life

	**Nail**	**DHS**	***p*-Value**
**Number of patients**	29	28	
**Age, mean ± SD**	85.7 ± 5.5	84.1 ± 9.1	0.428
**Female, n(%)**	23 (79.3%)	23 (82.1%)	0.786
**ASA, n(%)**			0.093
2	8 (27.6%)	9 (32.1%)	
3	16 (55.2%)	19 (67.9%)	
4	5 (17.2%)	0	
**Charlson comorbidity index, mean±SD**	5.36 ± 1.3	5.1 ± 1.6	0.445
**Pre-op Hb (g/L), mean ± SD**	119.2 ± 15.6	118 ± 12.6	0.750
**Hb < 100g/L, n(%)**	4 (13.8%)	1 (3.6%)	0.352
**Cognitive status**			1.000
AMTS ≥ 8	18 (62.1%)	18 (64.3%)	
AMTS < 8	11 (37.9%)	10 (35.7%)	
**Pre-injury residential status, n(%)**			0.619
Home	23 (79.3%)	23 (82.1%)	
With family	-	1 (3.6%)	
Residential home	4 (13.8%)	4 (14.3%)	
Nursing home	2 (6.9%)	-	
**Pre-injury mobility status**			0.329
No aids	10 (34.5%)	8 (28.6%)	
With stick	8 (27.6%)	12 (42.6%)	
With frame	11 (37.9%)	6 (21.4%)	
Unknown	-	2 (7.1%)	
**Pre-injury-, median(range)**			
LEM	59.5 (31.3–100)	67.3 (48.3–93.8)	0.309
LHS	0.66 (0.53–1.00)	0.73 (0.54–1.00)	0.554
DEMQOL	87.0 (69–95)	73.6 (40–108)	0.382
DEMQOL (carer)	99.2 (84–117)	97.6 (64–115)	0.693

Table S2: Mortality rates as per AMTS group at 2, 4, 12 weeks and at 1 year.

**Mortality Rates, n(%)**	**AMTS ≥ 8 n = 36**	**AMTS < 8 n = 21**	***p*-Value**
**2 weeks**	0 (0%)	3 (14.3%)	**0.046**
**4 weeks**	0 (0%)	4 (19%)	**0.015**
**12 weeks**	0 (0%)	6 (28.6%)	**0.002**
**1 year**	4 (11.1%)	10 (50%)	**0.003**
